# Case report: atypical, unilateral optic nerve infiltration as the first sign of acute lymphoblastic leukemia (ALL) relapse

**DOI:** 10.1186/s12886-022-02421-y

**Published:** 2022-04-27

**Authors:** Haidar Khalil, Clemens Strohmaier, Matthias Bolz

**Affiliations:** Department of Ophthalmology and Optometry, Johannes Kepler University, 4020 Linz, Austria

**Keywords:** Case report, Optic nerve, Acute lymphoblastic leukemia, Relapse

## Abstract

**Background:**

We describe a case of an atypical presentation of leukemic optic nerve infiltration.

**Case presentation:**

A patient with acute lymphoblastic leukemia (ALL) in remission suffered from sudden right eye vision loss. At the time of presentation, the affected eye presented with an afferent pupillary defect, while the fundus examination was normal. A complete work up of the patient revealed no signs of ALL relapse, but MR imaging of the optic nerve showed contrast agent uptake consistent with optic nerve infiltration. The patient developed a fulminant ALL relapse and died shortly after. Histology of the optic nerve showed a leukemic infiltration with CD10 positive cells.

**Conclusions:**

This is the first report of an ALL relapse in the optic nerve without intraocular signs. Patients’ medical history should therefore be taken into consideration in patients with unclear vision loss.

## Background

Acute lymphoblastic leukemia (ALL) is a malignant hematologic disease presenting clinically with proliferation of immature lymphatic cells in the bone marrow and peripheral blood [1]. Ocular symptoms can occur in different stages of ALL. They can be the first manifestation of leukemia (either primary or secondary), occur after the diagnosis has been established, or be the first sign of a relapse after remission [2]. Secondary ocular manifestations include retinal hemorrhages and retinal venous occlusions due to hematologic abnormalities [2]. Primary ocular leukemic infiltration can also affect the orbit, anterior segment, and the uvea. Leukemic infiltration of cranial nerves can cause palsies with motility disorders or optic neuropathy with sudden vision loss, similar to the presentation in our case [3]. However, previous reports have shown intraocular involvement with infiltration of the optic nerve and associated optic disc swelling and fundus hemorrhages [4–9].

We describe a case of an atypical presentation of leukemic optic nerve infiltration. This is the first report of an ALL relapse in the optic nerve without any intraocular signs.

## Case presentation

**Fig. 1:**
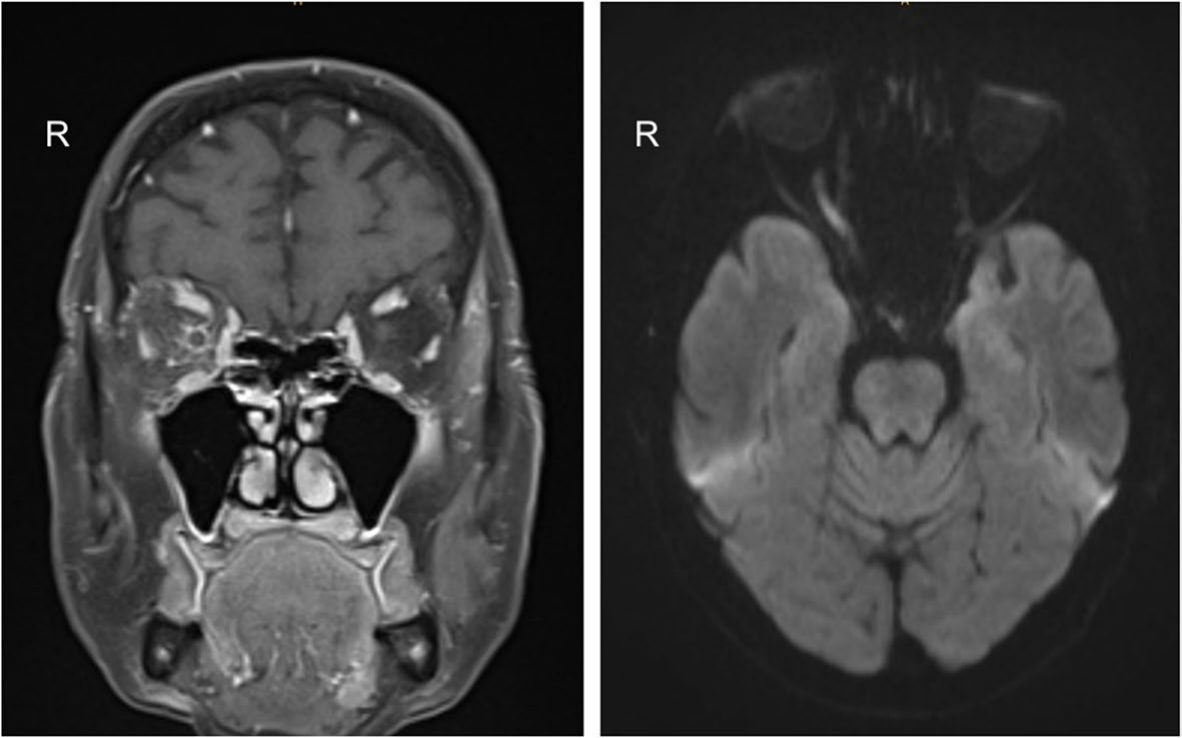
Left image: Coronal section of the orbital apex. Surrounding the right optic nerve, pronounced gadolinium uptake can be seen, consistent with perineural infiltration. Right image: Horizontal section of the optic nerves. The right optic nerve exhibits gadolinium uptake over most of its intraorbital course

**Fig. 2:**
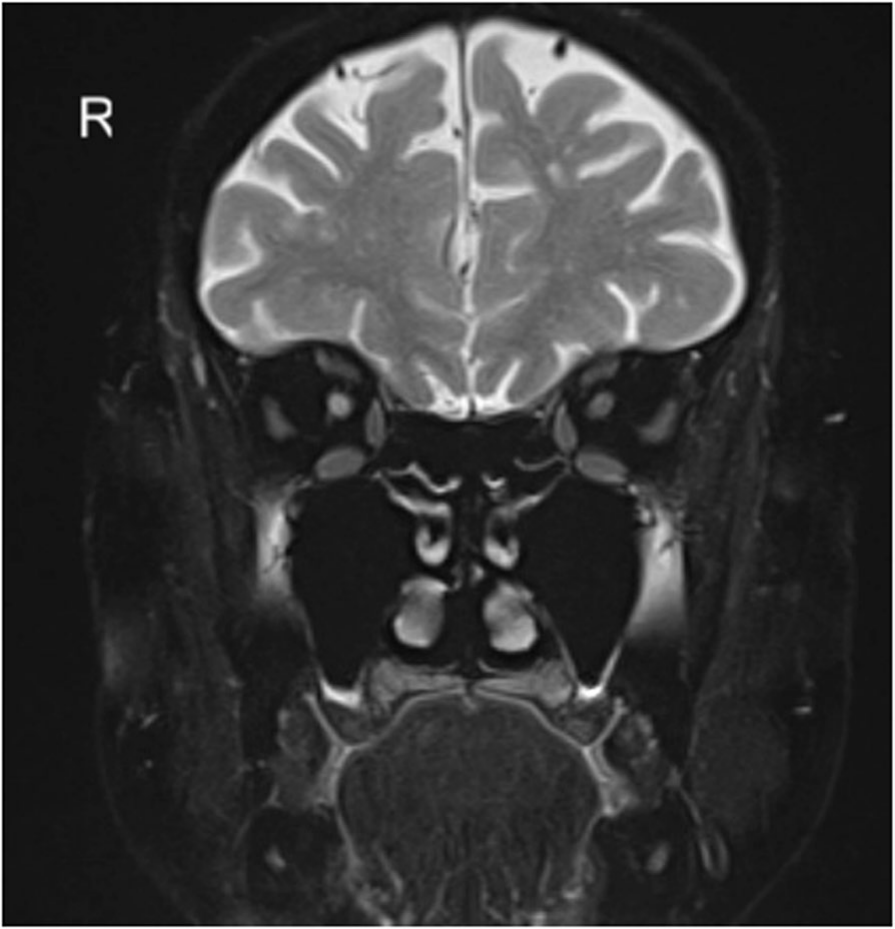
MR imaging with T2 weighted coronal section of the orbit without enhancement. Signal alteration in the right optic nerve can be seen

A 73-year-old woman presented with sudden decreased visual acuity in the right eye after the fourth cycle of intravenous blinatumomab therapy for acute lymphoblastic leukemia (common ALL, CD10/20 positive). At the time of presentation, her ALL status was complete remission with normal laboratory tests for 12 months. Ocular status in both eyes showed a clear cornea, deep anterior chamber (AC), no cells and no flare in the AC, and a mild nuclear cataract. Intraocular pressure was 12 mmHg in the right eye and 14 mmHg on the left eye. Funduscopic examination showed a vital optic nerve in both eyes with no signs of swelling or a difference in color. No retinal abnormalities or hemorrhages were detected. Optic coherence tomography of the macula showed proper retinal architecture and no pathology. Visual acuity (VA) in the right eye was finger counting and in the left eye 20/25. In the right eye, an abnormal relative afferent pupillary deficit (RAPD) could be detected during a swinging flashlight test, whereas the left eye exhibited normal responses. Although fundus examination was normal in the presented case, an ALL relapse was suspected because of the patient´s medical history. A complete oncologic and ophthalmologic re-assessment of the patient, including lumbar puncture, MRI, PET-computer tomography, and visually evoked potentials (VEP) were performed. VEP examination showed no response in the right eye and a regular response in the left eye. Brain MRI showed enhanced uptake of gadolinium in the prechiasmatic region of the right optic nerve and in the optic nerve sheath. Furthermore, unspecific leukoencephalopathy was detected (see Figs. 1 and 2). PET-CT revealed decreased metabolism of the right optic nerve compared to the left optic nerve. CSF (cerebrospinal fluid) exam (performed within 2 weeks of the initial presentation) revealed limited cells which were scattered lymphocytes with no sign of activation. Blood counts showed a mild increase in hematocrit and monocytes, but the rest of the blood differential count was unremarkable. Subsequently, direct toxicity from intravenous blinatumomab was also considered. However, the patient then developed a fulminant ALL relapse 1 month later and died of pneumonia and septic shock during intensive care. Autopsy revealed infiltration of the optic nerve with CD10 positive cells, consistent with an ALL relapse in the optic nerve.

## Discussion and conclusions

Untreated ALL in adult patients has very poor prognosis, but therapy has improved significantly in the last decades raising long-term survival rates [10]. Relapse of ALL is described by the reappearance of leukemia cells in peripheral blood or bone marrow after a complete remission and occurs often within the first two years after diagnosis [11]. Symptoms of leukemia are non-specific and can include night sweats, fever, weight loss, fatigue, recurrent infections, and/or involvement of the central nervous system including meningeal symptoms.

Blinatumomab is a bispecific antibody binding simultaneously to CD19 on the surface of B-cells and CD3 on the surface of T-cells leading to tumor cell lysis [12]. Compared to standard chemotherapy, Blinatumomab has shown promising results regarding long term survival in a randomized, multi-center trial [13] and is approved for the treatment of B-cell ALL [12]. Box warnings of Blinatumomab include neurological symptoms such as encephalopathy, convulsions, speech disorders, delirium, and balance disorders. Also, neurological toxicities, which may be life threatening are mentioned. Sudden vision loss or other ophthalmological side effects are not mentioned in the warnings and have not been described in the current literature.

Since the diagnostic blood work-up did not reveal an ALL relapse, a direct intravenous Blinatumomab toxicity was considered, although it has not described in the current literature. Further, this patient presented with vision changes twelve months after the last Blinatumomab injection. Still, leukemic infiltration of the optic nerve usually presents clinically as optic nerve head edema and with retinal hemorrhages. In the literature, few reports of optic nerve infiltration as the first sign of acute lymphatic leukemia relapse have been published and all reported significant intraocular involvement [4–9]. Retinal angiography and ultrasound of the eye was not performed initially since the funduscopic examination showed no pathologic signs. Therefore, retrobulbar involvement as the cause for the optic neuropathy was suspected. We transferred the patient to the oncologic department of our hospital to rule out an ALL relapse. Therefore we did not perform retinal angiography and ultrasound, even though it is a sensitive diagnostic modality for recognizing ocular involvement of acute leukemia [9]. The optic nerve may serve as a “sanctuary “ for lymphoblastic blast cells and therapy might not clear all tumor cells, leading to late relapse [14]. Also the blood brain barrier might lead to reduced penetration of systemic ALL treatment to the optic nerve [15]. During follow-up, the patient developed a severe pneumonia and was admitted to the intensive care unit. She died shortly after admission due to septic shock. Autopsy revealed a leukemic optic nerve infiltration with CD-10 positive cells. In conclusion, an atypical presentation of retrobulbar optic nerve infiltration should be considered in ALL patients with a sudden loss and a normal fundus exam.

## Data Availability

All data generated or analyzed during this study are included in this published article.

## Abbreviations

ALL: Acute lymphoblastic leukemia
VA: Visual acuity
OU: Oculus uterque
OD: Oculus dexter
MR: Magnetic resonance
PET-CT: Positron emission tomography – computed tomography
CSF: Cerebrospinal fluid

## References

[1] Medinger M, Heim D, Lengerke C, Halter JP, Passweg JR (2019). Acute lymphoblastic leukemia - diagnosis and therapy. Ther Umsch.

[2] Vishnevskia-Dai V, Sella King S, Lekach R, Fabian ID, Zloto O (2020). Ocular Manifestations of Leukemia and Results of Treatment with Intravitreal Methotrexate. Sci Rep.

[3] Reddy SC, Jackson N, Menon BS (2003). Ocular involvement in leukemia–a study of 288 cases. Ophthalmologica.

[4] Pflugrath AE, Brar VS (2020). Bilateral optic nerve and retinal infiltration as an initial site of relapse in a child with T-cell acute lymphoblastic leukemia. Am J Ophthalmol case reports.

[5] Fadilah SAW, Raymond AA, Cheong SK, Faridah A (2001). Relapsed Acute Lymphoblastic Leukemia Presenting as Optic Neuritis. Hematology.

[6] Wong BJ, Berry JL (2017). Acute Lymphoblastic Leukemia Relapse Presenting as Optic Nerve Infiltration. JAMA Ophthalmol.

[7] Shor N, Fardeau C, Bonnin S (2019). Teaching NeuroImages: Optic and third cranial nerves infiltration as initial relapse of acute lymphoblastic leukemia. Neurology.

[8] Chawla B, Agarwal P, Tandon R, Titiyal JS (2009). Peripheral ulcerative keratitis with bilateral optic nerve involvement as an initial presentation of acute lymphocytic leukemia in an adult. Int Ophthalmol.

[9] Camera A, Piccirillo G, Cennamo G (1993). Optic nerve involvement in acute lymphoblastic leukemia. Leuk Lymphoma.

[10] Bassan R, Hoelzer D (2011). Modern therapy of acute lymphoblastic leukemia. J Clin Oncol.

[11] Fielding AK, Richards SM, Chopra R (2007). Outcome of 609 adults after relapse of acute lymphoblastic leukemia (ALL); an MRC UKALL12/ECOG 2993 study. Blood.

[12] Lau KM, Saunders IM, Goodman AM (2021). Characterization of relapse patterns in patients with acute lymphoblastic leukemia treated with blinatumomab. J Oncol Pharm Pract.

[13] Kantarjian H, Stein A, Gökbuget N (2017). Blinatumomab versus Chemotherapy for Advanced Acute Lymphoblastic Leukemia. N Engl J Med.

[14] Ninane J, Taylor D, Day S (1980). The eye as a sanctuary in acute lymphoblastic leukaemia. Lancet (London, England).

[15] da Costa DR, Fernandes RD, Susanna FN, da Silva Neto ED, Monteiro MLR (2021). Complete reversal of bilateral optic nerve infiltration from lymphoblastic leukemia using chemotherapy without adjuvant radiotherapy. BMC Ophthalmol.

